# Differences in Motion Accuracy of Baduanjin between Novice and Senior Students on Inertial Sensor Measurement Systems

**DOI:** 10.3390/s20216258

**Published:** 2020-11-02

**Authors:** Hai Li, Selina Khoo, Hwa Jen Yap

**Affiliations:** 1 Centre for Sport and Exercise Sciences, University of Malaya, Kuala Lumpur 50603, Malaysia; 10000861@njtc.edu.cn (H.L.); selina@um.edu.my (S.K.); 2 College of Sport, Neijiang Normal University, Neijiang 641112, China; 3 Department of Mechanical Engineering, Faculty of Engineering University of Malaya, Kuala Lumpur 50603, Malaysia

**Keywords:** motion capture, inertial sensor measurement systems, motion accuracy, Baduanjin, physical education

## Abstract

This study aimed to evaluate the motion accuracy of novice and senior students in Baduanjin (a traditional Chinese sport) using an inertial sensor measurement system (IMU). Study participants were nine novice students, 11 senior students, and a teacher. The motion data of all participants were measured three times with the IMU. Using the motions of the teacher as the standard motions, we used dynamic time warping to calculate the distances between the motion data of the students and the teacher to evaluate the motion accuracy of the students. The distances between the motion data of the novice students and the teacher were higher than that between senior students and the teacher (*p* < 0.05 or *p* < 0.01). These initial results showed that the IMU and the corresponding mathematical methods could effectively distinguish the differences in motion accuracy between novice and senior students of Baduanjin.

## 1. Introduction

Traditional Chinese sport has been a compulsory component of Physical Education (PE) in universities in China since 2002 [1]. Although there are various traditional Chinese sports to choose from, 76.7% of universities taught martial arts in their PE curriculum [2]. In 2016, the Communist Party of China and the Chinese government adopted the ‘Healthy China 2030’ national health plan [3]. In this plan, Baduanjin was identified as a traditional Chinese sport that was promoted and supported by the government. This resulted in increased Baduanjin teaching and research in universities throughout the country [4].

Although universities in China must incorporate traditional Chinese sports into their PE curriculum, there have been problems with its implementation. These include a high student-teacher ratio, uninteresting forms of teaching-learning resources, and an incomplete assessment system. These three problems adversely affected the requirements for teaching quality set by the People’s Republic of China Ministry of Education [5,6]. Although the high student–teacher ratio has been a problem since 2005, it has yet to be resolved [7,8]. Teachers are not able to provide individual guidance to each student because of the large number of students in the class. As a result, teachers cannot correct all the students’ mistakes, and students are not aware of their incorrect movements [9]. 

In recent years, motion capture (Mocap) has been widely applied in fields such as clinical and sports biomechanics to distinguish between different types of motions or analyze differences between motions [10,11]. Studies have also applied Mocap in PE for adaptive motion analysis to evaluate the motion quality of learners and feedback the information to assist them in detecting and correcting their inaccurate motions. In the study by Koji Yamada et al. [12], a system based on Mocap was developed for Frisbee learners. Researchers used the Kinect device to obtain 3D motion data of learners during exercise, detect their pre-motion/motion/post-motion, and display the feedback information to improve their motions. The results showed that the system developed by the researchers can effectively improve the motions of learners [12]. Chen et al. [13] applied Kinect in Taichi courses in universities. The 3D data of motions from novice students captured by Kinect were compared with an expert in order to evaluate the quality of motions and students were informed of their results. The research showed that the motion evaluation system on Kinect developed by the researchers accelerated learning by novice Taichi students. More recently, Amani Elaoud et al. [14] used Kinect V2 to obtain red, green, blue and depth (RGB-D) data of motion. They used these data to compare the differences between novice students and experts on the central angles of points that affect throwing performance in handball. These experiments show how researchers have used various categories of Mocap.

Based on different technical characteristics, the application of Mocap in PE can be divided into four categories: optoelectronic system (OMS) [15], electromagnetic system (EMS), image processing systems (IMS), and inertial sensor measurement system (IMU) [16]. In these four Mocap categories, OMS is the most accurate and is considered to be the gold standard in motion capture [16,17]. However, OMS requires a large number of high-precision and high-speed cameras that will inevitably result in issues related to cost, coordination, and manual use [18]. Moreover, OMS cannot capture the movement of objects when the marker is obscured [19]. These deficiencies have limited the practical application of OMS in PE. The advantage of EMS over OMS is that it can measure motion data of a specific point of the body regardless of visual shielding [20]. However, EMS is susceptible to interference from the electromagnetic environment which distorts measurement data [21]. Also, EMS has to be kept within a certain distance from the base station, which limits the use range [22]. IMS has better accuracy compared to EMS and an improved range compared to OMS [16]. Most studies have used low-cost IMS (such as the Kinect device) to capture motion for analyzing motion in PE. However, there are some disadvantages in low-cost IMS, namely low-accuracy, insufficient environment adaptability, and limited range of motion because the Kinect sensor has a small field of vision [16]. High-performance IMS does not have these shortcomings. Generally, high-performance IMS has favorable accuracy and a good measurement range. However, high-performance IMS requires expensive high quality and/or high-speed cameras which has limited its application [16]. 

Based on the disadvantages of low-cost IMS in its application in PE, applying IMU (a motion capture consisting of an accelerometer, gyroscope, and a magnetometer) in PE may mitigate these application problems [23]. In recent years, the development of technology has reduced the cost of IMU, making it possible to be used in PE. The validity of assessing motion accuracy of IMUs has been confirmed. Poitras et al. [24] confirmed the criterion validity of a commercial IMU system (MVN Awinda system, Xsens) by comparing it to a gold standard optoelectronic system (Vicon). Compared to low-cost IMS, IMU has certain advantages in environmental adaptability and a sufficient range of motion. The IMU does not require any base station to work, which means it is the most mobile of the available motion capture systems [16]. Moreover, IMU can measure high-speed movements and is non-invasive for the user, making it an attractive application for PE [16,25].

However, there are a few issues capturing motions using IMU. First, the IMU sensors are sensitive to metal objects nearby which distort the measurement data [16]. Therefore, participants should wear fewer metal objects when capturing motion using IMU. Fortunately, in traditional Chinese martial arts such as Baduanjin, exercisers should, in principle, wear traditional Chinese costumes without any metal, which minimizes the impact of metals on IMU sensors. Second, a common IMU system, Perception Neuron 2.0, was used in our research which uses data cables to connect all sensors with a transmitter. Although users cannot wear this wired IMU system on their own, it does not affect the accuracy of the data. Also, the latest IMU overcomes this problem that users can’t wear it on their own by having each sensor transmit the data to the external receiving terminal directly [26]. 

Therefore, we propose applying IMU in Baduanjin by developing a system that assesses and records the quality of motions to assist teachers and students in determining inaccurate motions. Using IMU, students can learn Baduanjin independently after class and teachers can evaluate students’ progress, which is useful for formative assessment. That may alleviate current problems faced in PE classes in Chinese universities. For this purpose, we explored the feasibility of using an IMU to distinguish the difference in motion accuracy of Baduanjin between novice and senior students.

## 2. Materials and Methods

### 2.1. Overview

**Figure 1:**
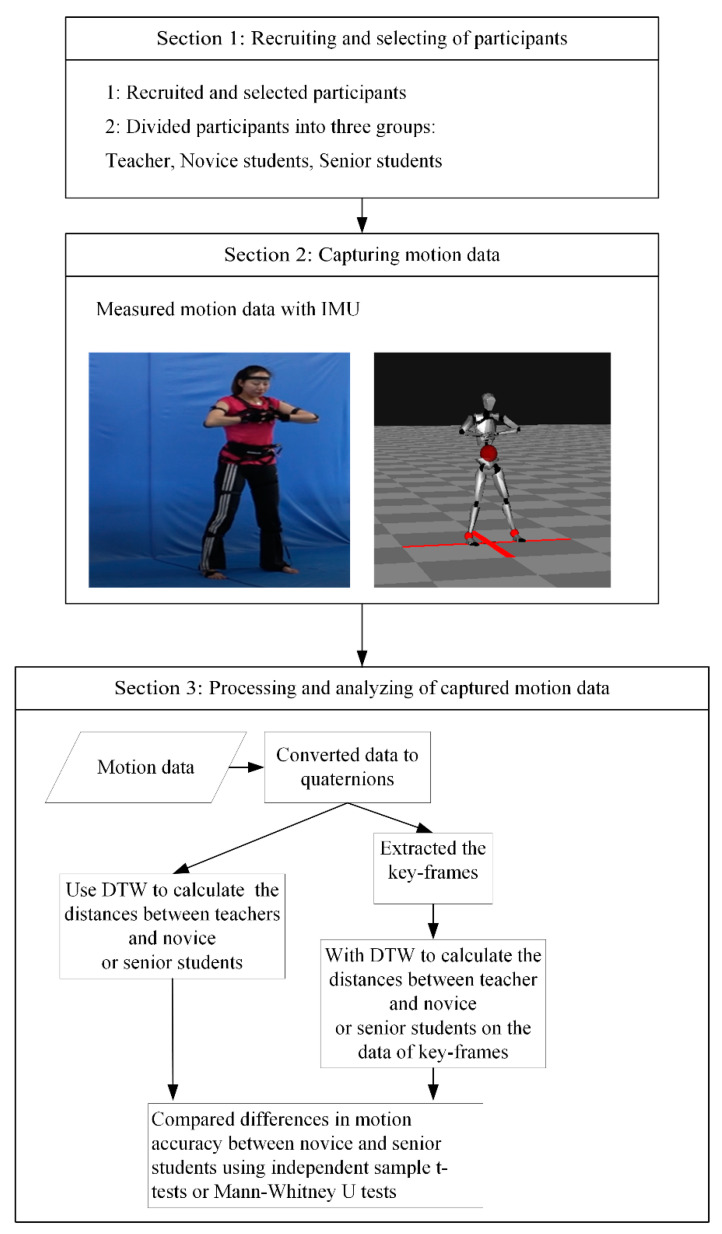
Flow diagram of the study.

This study consists of three sections, namely recruiting and selecting participants, capturing motion data of Baduanjin participants on IMU, and processing and analyzing the motion data. We invited teachers and students from a university in Southwest China to participate in the study. We divided them into three groups—teachers, novice students, and senior students. We captured motion data of all participants on IMU when they practised Baduanjin. The motion data were converted to quaternions and analysed in two different ways. The first way was based on the quaternions of motion, where dynamic time warping (DTW) was used to calculate the distances between the quaternions of the teacher and the two groups of students (novice and senior). The motion accuracy of the students was expressed by distances. DTW is a classic similarity method to solve the time-warping issue in similarity computation of time series [27]. Compared with the other methods, namely the hidden Markov model (HMM) and symbolic aggregate approximation (SAX), the taken time of DTW is shorter [28,29]. Considering that, in the actual teaching, students need to get feedback information and a large number of student data in realtime, we adopted DTW in the study. The second way used the extracted key-frames to calculate distances. Based on the quaternions of key-frames, DTW was used to calculate the distances. Finally, based on data of the distances, an independent sample T-test or Mann–Whitney U test was used to define whether the motions of the two groups of students (novice and senior) were different in motion accuracy (see Figure 1).

### 2.2. Recruiting and Selecting Participants

In this study, we invited a martial arts PE teacher and undergraduate students to participate in the study. The inclusion criteria for participants are as follows:

Teacher: martial arts PE teacher, former national martial arts athlete, with an undergraduate and master’s degree in traditional Chinese sports (martial arts specialization), and more than ten years’ experience teaching Baduanjin.

Novice students: undergraduate students in the university with no experience of Baduanjin, without a disability and no clinical or mental illness.

Senior students: undergraduate students in the university who have passed Baduanjin in their PE course, without a disability, and no clinical or mental illness.

Participants read the information sheet that outlined the purpose and procedure of the study. Those who agreed to participate were given the consent form to sign. 

### 2.3. Capturing Motion Data of Participants on IMU

**Figure 2:**
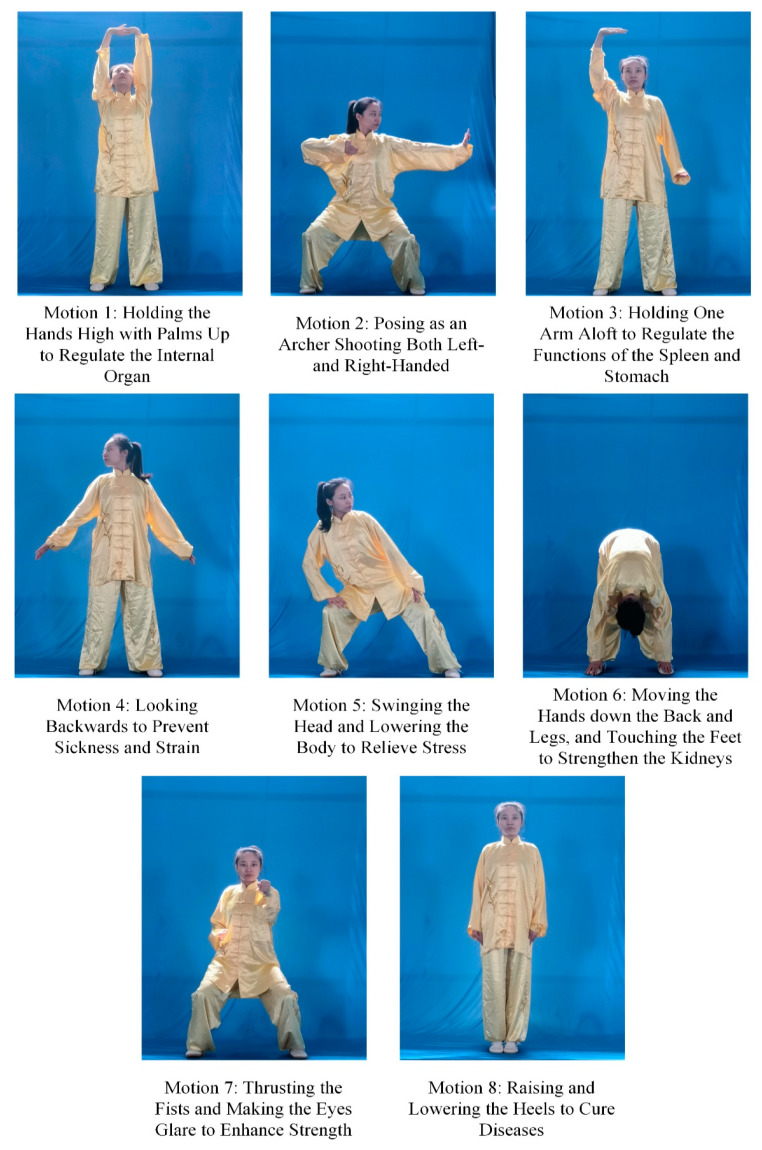
Eight standard motions of Baduanjin.

Baduanjin is a traditional Chinese martial art for fitness. The speed of motions is relatively slow [30]. We used IMU to capture the motion data of the teacher, novice, and senior students for eight standard motions of Baduanjin as shown in Figure 2.

#### 2.3.1. IMU

We used Perception Neuron 2.0, a low-cost IMU developed by Noitom, to capture Baduanjin motion data of participants [31]. This IMU includes 17 inertial sense units and each unit comprised a 3-axis gyroscope, 3-axis accelerate, and 3-axis magnetometer, which measures and records the rotation angle data of 17 position points of human movement. Sers et al. [32] compared the IMU used in our study with a gold standard optoelectronic system (Vicon), and confirmed the IMU’s effectiveness in measuring motion accuracy. The supporting software of the IMU, Axis neuron software developed by Noitom, transforms the recorded data into Biovision Hierarchy (BVH) motion files.

#### 2.3.2. Capturing Motion Data

Before measuring the motion data, the teacher and senior students practised Baduanjin for 30 min. As the novice students had not learned Baduanjin, they followed the demonstration of the teacher practising Baduanjin for 30 min. After the practice, the motion data of participants were measured by IMU. No feedback was given to students during practice. 

### 2.4. Data-Analysis

#### 2.4.1. Extracting and Converting Raw Data

**Figure 3:**
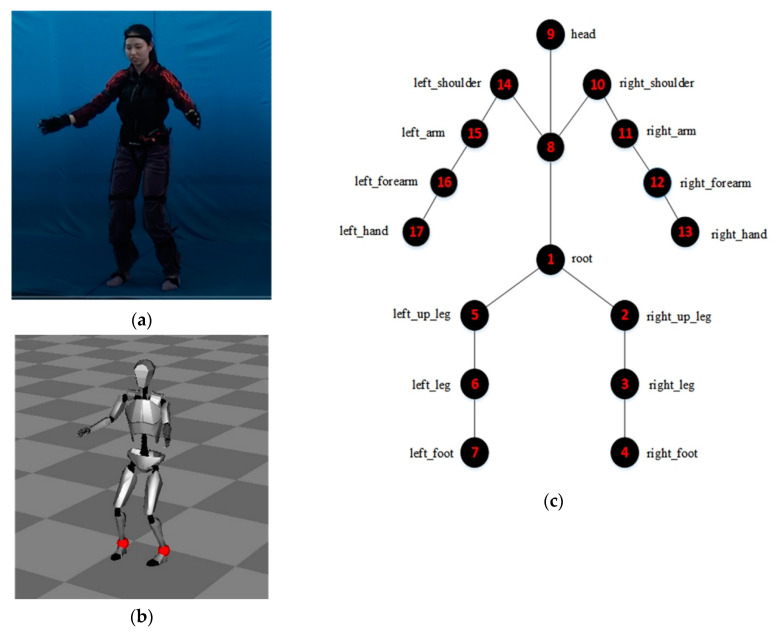
The skeleton hierarchy information of BVH on the IMU (Perception Neuron 2.0): (**a**) A participant wearing the IMU (Perception Neuron 2.0) to measure the motion data; (**b**) The interface of Perception Neuron 2.0; and (**c**) The skeleton model of BVH file for Perception Neuron 2.0.

The raw data was converted into BVH file by the Axis neuron software. The BVH file is a file format developed by the BVH Company to store skeleton hierarchy information and three-dimensional motion data [33]. The BVH file comprises two parts: one is used to store skeleton hierarchy information and the other to store motion information. The skeleton hierarchy information includes the connection relationship between joint points and the offsets of the child joint points from their parent skeleton points. In the skeleton hierarchy, the first skeleton point is defined as Root. Root is the parent of all other skeleton points in the skeleton hierarchy. Motion information stores the global translation amount and the rotation amount of Root in each frame of the movement. The global translation amount is the position coordinate: X position, Y position, and Z position in the world coordinate system and the rotation amount is the rotation component: X rotation, Y rotation, and Z rotation in the Euler angle [33]. The motion information of other skeleton points is recorded on the rotation amount related to the parent points. The IMU used 17 sensors to measure motion data on 17 points of the body and the recorded order of the rotation amount of each point is Z rotation, Y rotation, and X rotation. The skeleton hierarchy information of BVH on the IMU and the skeleton model are shown in Figure 3.

In the BVH file, the rotation data is recorded on the Euler angle of 17 skeleton points. Some issues with rotation data expressed on the Euler angle (gimbal lock and singularity problems) were overcome using quaternion [34]. Quaternion is a 4-dimensional hyper-complex number, expressing a three-dimensional vector space on real numbers [35]. We used four-tuple notation to represent quaternion as follows:

(1) q=[w,x,y,z]

In this quaternion, *w* is the scalar component, and *x*, *y*, *z* are the vectors. 

Therefore, the format of the rotation data from BVH files was converted from Euler angle to quaternion. If the order of rotation in Euler angle is *z*, *y*, *x*, we used α, β, γ to represent the rotation angles of the object around *x*, *y*, and *z* axes. The corresponding quaternion can be converted as follows:

(2) $$\begin{array}{cc} q & =\left[\begin{array}{l} w\\ x\\ y\\ z \end{array}\right]=\left[\begin{array}{l} \cos(\gamma /2)\\ 0\\ 0\\ \sin(\gamma /2) \end{array}\right]\left[\begin{array}{l} \cos(\beta /2)\\ 0\\ \sin(\beta /2)\\ 0 \end{array}\right]\left[\begin{array}{l} \cos(\alpha /2)\\ \sin(\alpha /2)\\ 0\\ 0 \end{array}\right]\\ & =\left[\begin{array}{c} \cos(\gamma /2)\cos(\beta /2)\cos(\alpha /2)+\sin(\gamma /2)\sin(\beta /2)\sin(\alpha /2)\\ \cos(\gamma /2)\cos(\beta /2)\sin(\alpha /2)-\sin(\gamma /2)\sin(\beta /2)\cos(\alpha /2)\\ \cos(\gamma /2)\sin(\beta /2)\cos(\alpha /2)+\sin(\gamma /2)\cos(\beta /2)\sin(\alpha /2)\\ \sin(\gamma /2)\cos(\beta /2)\sin(\alpha /2)-\cos(\gamma /2)\sin(\beta /2)\sin(\alpha /2) \end{array}\right] \end{array}$$

#### 2.4.2. Extracting Key-Frames

After extracting the motion data, we used key-frames extraction to reduce the motion data. Due to the limited storage and bandwidth capacity available to users, the large amount of motion data collected on Mocap may restrict its application [36]. Key-frames extraction, which extracts a small number of representative key-frames from a long motion sequence, is widely used in motion analysis. This technology can reduce the data amount, which facilitates data storage and subsequent data analysis [36,37]. 

##### Extraction of Key-Frames on Inter-Frame Pitch

We used the distance between quaternions to evaluate the inter-frame pitch between frames and set a threshold of inter-frame pitch to extract key-frames [38]. The method is based on the rotation data of each skeleton point which is represented as a quaternion and uses a simple form to evaluate the distance between two quaternions. The inter-frame pitch between the two frames is assessed by the sum of the distances between the quaternions of every point. The process is constructed with three sections: calculating the distance between quaternions, calculating the inter-frame pitch between frames, and extracting key-frames on the set threshold of inter-frame pitch.

1. The distance between quaternions

To evaluate the distance between two quaternions, the conjugate quaternion q* of a quaternion is defined as follows:

(3) q*=[w,−x,−y,−z]

and the quaternion norm ||*q*|| is defined as follows:

(4) $$\left\|q\right\|=\sqrt{w^{2}+x^{2}+y^{2}+z^{2}}$$

then:

(5) $${\left\|q\right\|}^{2}=qq*=w^{2}+x^{2}+y^{2}+z^{2}$$

when a quaternion norm ||*q*|| is 1, which means:

(6) $$w^{2}+x^{2}+y^{2}+z^{2}=1$$

the quaternion is a unit quaternion. A quaternion is converted to a unit quaternion by dividing it by its norm.

From the definitions of conjugate quaternion, quaternion norm, and unit quaternion, we can define the inverse of a quaternion (*q*^−1^) as follows [39]:

(7) $$q^{-1}=\frac{1}{\left\|q\right\|}=\frac{1}{{\left\|q\right\|}^{2}}q*,\;\left\|q\right\|\neq 0$$

According to Shunyi et al. [38], if there are two quaternions: *q*_1_, *q*_2_ are unit quaternions and:

(8) $$q_{1}q_{2}{}^{-1}=\left[w,\;x,\;y,\;z\right]$$

the distance between the quaternions *q*_1_ and *q*_2_ is:

(9) $$d(q_{1},q_{2})=\arccos w$$

Therefore, we converted the rotation of a skeleton point based on Euler angles into quaternion, then normalized and converted the quaternion into unit quaternion, and finally calculated the difference between any two quaternions of the point according to Equation (9).

2. Calculation of Inter-Frame Pitch between Two Frames

We used the sum of the differences between the quaternions at 17 skeleton points to evaluate the inter-frame pitch between two frames. The human motion represented by the BVH file are discrete-time vectors, which is the same after conversion to quaternions [38]. The weightage for different points needs to be taken into account when calculating the inter-frame pitch due to the tree-structure (parent-child) of the BVH format. Referring to the methods used in previous research [38,40], and the relationship structure between the skeleton points on the IMU in this study (see Figure 3), we assigned the weightage values of the 17 skeleton points as shown in Table 1.

If *t*_1_ and *t*_2_ are the two frames in a sequence of frames, we defined the inter-frame pitch between two frames: *t*_1_ and *t*_2_ as the following equation:

(10) $$D(t_{1},t_{2})=\sum_{i=1}^{n}W_{i}d(q_{i}(t_{1}),q_{i}(t_{2}))$$

In Equation (10), *n* represents the total number of skeleton points (*n* = 17), *W_i_* represents the weightage of each skeleton point (shown in Table 1), and *q_i_* represents the quaternions of each skeleton point.

3. Key-frames extraction on the set threshold of inter-frame pitch

**Figure 4:**
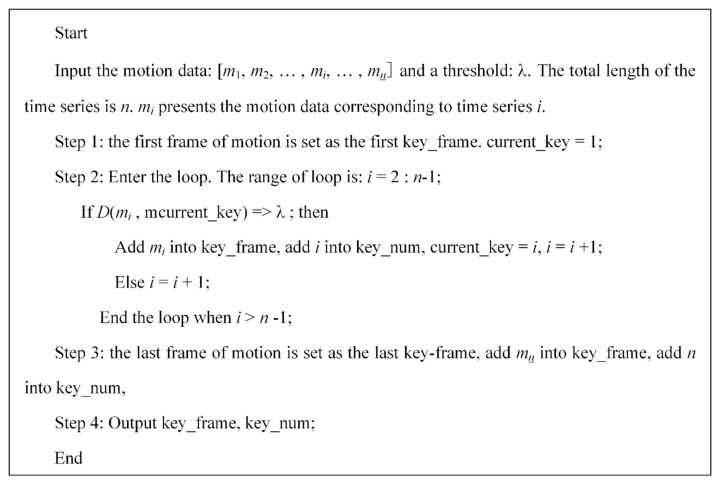
The algorithm steps of key-frames extraction.

Based on the inter-frame pitch between two frames, we set: key_frame as an array to store the quaternion corresponding to the key-frames of motion; key_num as a set of vector to store the serial number corresponding to a key-frame; key_num1 presents the time series number corresponding to the first key-frame; current_key as the last frame in the set of key_num. λ is a preset threshold value of inter-frame pitch which is mainly determined based on the demand for a compression rate of frames. The algorithm steps are shown in Figure 4.

4. Motion reconstruction error

The purpose of motion reconstruction is to rebuild the same number of frames as the original frames based on interpolation reconstruction of non-key-frames between adjacent key-frames [38,41]. First, individually, the position coordinates (in the world coordinate system) of points were calculated on the point hierarchy and relative rotation angle between the points in the BVH file. Second, given that *p_t_*_1_ and *p_t_*_2_ are the positions of a point of adjacent key-frames in time *t*_1_ and *t*_2_, then *p_t_* (representing the position of a point of non-key-frame in time *t*) is calculated by linear interpolation between *p_t_*_1_ and *p_t_*_2_ as follows [41]:

(11) $$\begin{array}{c} p_{t}=u(t)p_{t1}+(1-u(t))p_{t2},\\ u(t)=\frac{t_{2}-t}{t_{2}-t_{1}},\\ t_{1}<t<t_{2} \end{array}$$

**Figure 5:**
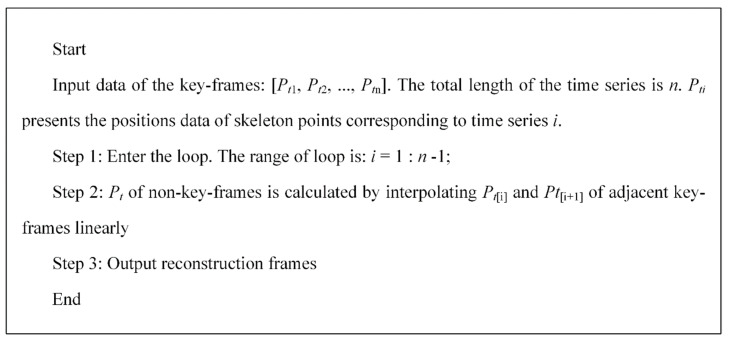
The algorithm steps of motion reconstruction.

The algorithm steps of motion reconstruction are shown in Figure 5.

In this study, we used the position error of the human posture to calculate the reconstruction error between the reconstructed frames and the original frames [38]. Assuming *m*_1_ is the original motion sequence, *m*_2_ is the reconstruction motion sequence from the key-frames, the reconstruction error *E*(*m*_1_, *m*_2_) is evaluated as [42]:

(12) $$E(m_{1},m_{2})=\frac{1}{n}\sum_{i=1}^{n}D(p_{1}^{i}-p_{2}^{i})$$

The distance of human posture is used to measure the position error of human posture:

(13) $$D(p_{1}^{i}-p_{2}^{i})=\sum_{k=1}^{m}{\left\|p_{1,k}^{i}-p_{2,k}^{i}\right\|}^{2}$$

In this equation, *m* represents the total number of skeleton points, $p_{1,k}^{i}$ is the position of *k* point in *i* frame of the original motion sequence, and $p_{2,k}^{i}$ is the position of *k* point in *i* frame in the reconstruction sequence.

##### Extraction of Key-Frames on Clustering

A problem with the key-frames extraction on inter-frame pitch is that the compression rate of the key-frames with the same inter-frame threshold for different actions may vary considerably [40]. As the eight motions of Baduanjin are quite different, the key-frames extraction on the inter-frame pitch may cause some motions to be compressed too much, and some motions not compressed enough. Therefore, we also chose another way to extract key-frames on clustering. This method was used for key-frames with the pre-set compression rate [43].

1. K-means clustering algorithm

K-means clustering algorithm is an iterative partition clustering algorithm. In this key-frame extraction method, we used the K-means clustering algorithm to cluster the 3D coordinates ([*x*, *y*, *z*]) of the skeleton points in the original frame. Assuming that the total length of the original frames is *N*, *i* represents the *i* frame in *N*. *p^i^* is the vectors of the 3D coordinate positions of all relevant skeleton points of the *i* frame in the original frames. Therefore, the vectors collection of the 3D coordinate data of every point of original frames is (*p*^1^, *p*^2^, …, *p^i^*), *p^i^* ∈ *R^N^*. According to the K-means clustering algorithm, the data of skeleton points (*R^N^*) in the frames is clustered into *K* (*K* ≤ *N*) clusters as follows [44]:

Step 1: Randomly select *K* cluster centroids from *R^N^* are *u*_1_, *u*_2_… *u_K_*;

Step 2: Repeat the following process to get convergence.

For the *p^i^* corresponding to one frame, we calculated the distances from each cluster centroid (*u_j_*, *j* ∈ *K*) and classified it into the class corresponding to the minimum distance [45]:

(14) $$D=\arg\min\sum_{i=1}^{N}\sum_{j=1}^{K}{\left\|p^{i}-u_{j}\right\|}^{2}$$

In this equation, *D* represents the minimum distance between the cluster centroid and the centre of *p^i^*, and when *D* is the smallest, *p^i^* is classified into class *j*.

For each class *j*, the cluster centroid (*u_j_*) of that class was recalculated:

(15) $$u_{j}=\frac{\sum_{i=1}^{N}r_{ij}p^{i}}{\sum_{i=1}^{N}r_{ij}}$$

In this equation, *r_ij_* indicates that when *p^i^* is classified as *j*, it is 1; otherwise, it is 0.

2. Key-frames extraction

Using the above k-means clustering algorithm, we extracted K cluster centroids from the original frame. Each cluster is clustered from the 3D coordinates of the 17 points in the original frames. Therefore, one cluster centroid is constructed with 51 (17 × 3) vectors. Based on these cluster centroids, we extracted the key-frames by calculating the Euclidean distance between the cluster centroid of each point and the corresponding point coordinates in the original frames. The steps to extract key-frames are as follows:

Start

Input the 3D coordinate data of every point of the original frames:

(16) $$\begin{array}{cc} \left(p^{1},p^{2}\ldots p^{i}\right),\; & p^{i}\in R^{N};\\ & p^{i}=(p_{1}^{i},p_{2}^{i}\ldots p_{j}^{i}),\;j=17;\\ & p_{j}^{i}=[x_{j}^{i},y_{j}^{i},z_{j}^{i}] \end{array}$$

and the number of key-frames to be extracted is *K*;

Step 1: Using the k-means clustering algorithm to calculate cluster centroids of the *K* clusters are expressed as:

(17) $$\begin{array}{l} u_{m}=(u_{m1},u_{m2}\ldots u_{mj}),\;m\in (1,2,3\ldots K),\;j=17;\\ u_{mj}=[x_{mj},y_{mj},z_{mj}] \end{array}$$

Step 2: Calculate the Euclidean distance of 3D coordinates between each point of the cluster and the corresponding point of the original frames:

(18) $$\begin{array}{cc} C_{m} & =\min(u_{m},p^{i})\\ & =\sum_{j=1}^{17}\min(dis(u_{mj},p_{j}^{i}));\\ dis(u_{mj,}p_{j}^{i}) & ={\left\|u_{mj}-p_{j}^{i}\right\|}^{2} \end{array}$$

$\min(dis(u_{mj},p_{j}^{i}))$ means that after calculating the distances between m cluster and all original frames, the *j* point of pi which value of $dis(u_{mj},p_{j}^{i})$ is minimum is recorded as 1; otherwise, it is recorded as 0. *i* of *p^i^* corresponding to the maximum value of *C_m_* is a sequence of key-frames.

Step 3: Sequences of key-frames are arranged from small to large after extraction. If the first frame and the last frame in the original frames are not included in the key-frames, the first frame and the last frame must be added into key-frames.

End

In this key-frames extraction, the number of key-frames can be preset. The key-frames of the corresponding compression rate is obtained by presetting the compression rate as follows [42]:

(19) K=c_rate*N

where *K* is the number of key-frames to be extracted, *c_rate* is the compression rate of the key-frame to be obtained, and *N* is the total number of original frames.

After extracting key-frames, we continued with the ways to motion reconstruction and evaluate reconstruction error as described above.

#### 2.4.3. Evaluate Motion the Accuracy of Motions Data

In this study, we referred to previous studies [13,46] to evaluate the motion accuracy of student motions by assessing the differences between students’ motions and teacher’s motions. Due to the difference in speed between individual movements, different time series were considered when assessing the difference between two motions. We chose DTW, a well-established method, to account for different time series to evaluate the difference in the motions between teachers and students [47]. Since DTW compares the other methods, i.e., HMM and SAX, without a training stage, the taken time is shorter. First, the derived quaternions were normalized in unit length of a quaternion: *q* = [*w*, *x*, *y*, *z*] can be described as: ||*q*|| = 1 and *w*^2^ + *x*^2^ + *y*^2^ + *z*^2^ = 1. Therefore, three components (*x*, *y*, *z*) out of the four components (*w*, *x*, *y*, *z*) of the quaternions can be used to represent the rotations of the skeleton points over a temporal domain. Then, we used DTW to evaluate the difference between two sequences of motions on the skeleton points. First, we assessed the difference between two motions on a single skeleton point. For example, there are two motion data on quaternions for a skeleton point from a teacher and a student, one from the teacher: *q_tea_*(*t*), one from a student: *q_stu_*(*t*). The length of the two sequences of quaternions are *n* and *m*:

(20) $$\begin{array}{l} q_{tea}(t)=q_{tea}(1),q_{tea}(2),\ldots ,q_{tea}(i),\ldots ,q_{tea}(n)\\ q_{stu}(t)=q_{stu}(1),q_{stu}(2),\ldots ,q_{stu}(j),\ldots ,q_{stu}(m) \end{array}$$

The vector in the quaternion arrays consists of three components (*x*, *y*, *z*) of quaternions. A distance matrix (*n* × *m*) is constructed to align the quaternions of two sequences. The elements (*i*, *j*) in the matrix represent the Euclidean distance: *dis*(*qtea*(*i*), *qstu*(*j*)) between the two points *q_tea_*(*i*) and *q_stu_*(*j*):

(21) $$dis(q_{tea}(i),q_{stu}(j))={\left|q_{tea}(i)-q_{stu}(j)\right|}^{2}$$

In the distance matrix, many paths are from the upper-left corner to the lower-right corner of the distance matrix. We used Φ*k* to represent any point on these paths: Φ*k* = (Φ*_tea_*(*k*), Φ*_stu_*(*k*)) where:

Φ*_tea_*(*k*): the value of *k* is 1, 2, …, *n*,

Φ*_stu_*(*k*): the value of *k* is 1, 2, …, *m*, 

Φ*k*, the value of *k* is 1, 2, …, *T*, (*T* = *n* × *m*)

We found a suitable path as the warping path, where the cumulative distance of path is the smallest of all paths [39]:

(22) $$DTW(q_{tea}(t),q_{stu}(t))=\min\sum_{k=1}^{T}dis(\Phi_{tea}(k),\Phi_{stu}(k))$$

Then, the distance of *DTW*(*qtea*(t),*q_stu_*(*t*)) is obtained through dynamic programming as follows [47]:

(23) $$\begin{array}{l} DTW(q_{tea}(t),q_{stu}(t))=f(n,m);\\ f(0,0)\;=\;0;\\ f(0,1)=f(1,0)=\infty ;\\ f(i,j)=dis(q_{tea}(i),q_{stu}(j))+\min\{f(i-1,j),f(i,j-1),f(i-1,j-1)\},(i=1,2,\ldots ,n;j=1,2,\ldots ,m) \end{array}$$

To prevent the wrong matching by excessive time warping, the warping path was constrained near the diagonal of the matrix by setting the global warping window for DTW [48,49]. In this study, the global warping window is set as 10 percent of the entire window span: 0.1 × max(*n*, *m*). The cumulative distance of the warping path represents the difference of rotation between teacher and student on the skeleton points is shown in Equation (22). Then, the macro difference between students’ motions and teacher’s motions was evaluated by taking the average of the cumulative distances of all the skeleton points as follows:

(24) $$D(m_{tea},m_{stu})=\frac{\sum_{i=1}^{n}DTW(q_{tea}^{i},q_{stu}^{i})}{n}$$

In this equation, *m*_tea_ represents the teacher motion sequence; *m*_stu_ represents the students’ motion sequence, *q^i^* is the vectors of the quaternion of *i* skeleton point in the two motion sequences, and the total number of skeleton points is *n*.

Finally, data of the differences were analysed using IBM SPSS Statistics 25.0 to assess if there were significant differences in the motion accuracy of the two groups of students (novice and senior students) on the whole and each point. We used the independent sample T-test on data with normal distribution and the Mann–Whitney U test on data with non-normal distribution.

## 3. Results

### 3.1. Demographic Characteristics of Participants

We recruited 21 participants for this study, including a martial arts teacher, nine undergraduate students who have not learned Baduanjin (novice students), and 11 undergraduate students who had completed the Baduanjin course (senior students). All participants gave their informed consent for inclusion before they participated in the study. The study was conducted in accordance with the Declaration of Helsinki, and the protocol was approved by the University of Malaya Research Ethics Committee (UM.TNC2/UMREC–558). The demographic characteristics of the students are shown in Table 2. For each mean duration of the eight motions shown in Table 3, we measured all participants three times with IMU, resulting in 63 motion data.

### 3.2. Differences in Motion Accuracy between Novice and Senior Students on Original Frames

Algorithms explained in the data analysis section were coded with Matlab R2018b. Independent sample T-tests and Mann–Whitney U tests were used to assess the differences in motion accuracy of novice and senior students. 

Before assessing macro differences, we assessed the normality of original frames data using the Shapiro–Wilk test (see Table 4).

From Table 4, we can see that the data of the groups on Motions 2, 3, and 4 were normally distributed (*p* > 0.05), whereas the others were not. Therefore, we assessed the differences in motion accuracy of Motions 2, 3, and 4 between novice and senior students using independent sample T-tests (see Table 5). The differences in the motion accuracy of other motions between novice and senior students were assessed using Mann–Whitney U tests (see Table 6).

From Table 5 and Table 6, we can see significant differences (*p* < 0.05 or *p* < 0.01) in motion accuracy of all eight motions between novice and senior students. The differences in motion accuracy between the teacher and senior students were lower than the differences in motion accuracy between the teacher and novice students. 

**Figure 6:**
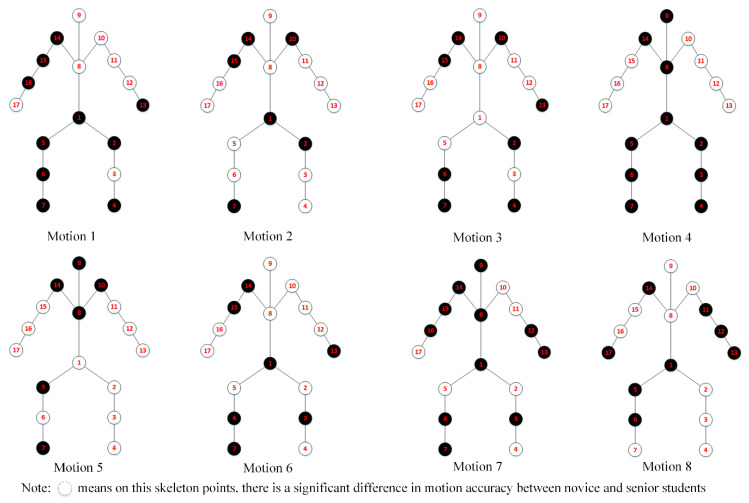
Differences in motion accuracy of points between novice and senior students on original frames.

We also evaluated the difference in motion accuracy on each skeleton point between novice and senior students (Figure 6).

From Figure 6, we found that out of the 17 points on eight motions of Baduanjin, there were significant differences in the motion accuracy between novice and senior students for some points. For example, in Motion 1, there were significant differences in motion accuracy between the two groups at the head and neck (points 8 and 9) and the right upper limb (points 10, 11, and 12).

### 3.3. Differences in Motion Accuracy between Novice and Senior Students on Key-Frames

#### 3.3.1. Compression Rate and Reconstruction Error of Two Different Key-Frames Extraction Methods

Motion accuracy is assessed based on key-frames. In this study, we chose two methods to extract key-frames. In the key-frames extraction method on inter-frame pitch, we selected different thresholds (0.1, 0.5, 1.0, 1.5, 2.0) to extract key-frames and evaluated the compression rate and the reconstruction error of corresponding key-frames on different thresholds. The results are shown in Table 7.

Table 7 shows significant differences in the compression rates of the different motions extracted under the same threshold. We can see when the threshold value is set to 1 for obtaining key-frames using the inter-frame pitch, there was a difference in average compression rates ranging from 7.08% to 20.78% for the eight motions of Baduanjin. Moreover, when the threshold value increased, the number of key-frames decreased, which decreased the compression rate. However, the error of motion reconstruction also increased. Based on the data in Table 7, it can be seen that in the five preset values, the compression rate and reconstruction error of the extracted key-frames are relatively reasonable when the threshold is 1. In the other key-frames extraction method on clustering, we chose different compression rates (5, 10, 15, 20, 25) to extract key-frames and evaluate the reconstruction error on different key-frames. The results are shown in Table 8.

From Table 8, we can see that as the compression rate increases, the error of motion reconstruction decreases. When the compression rate increased from 5% to 15%, the reconstruction error dropped sharply. But when the compression ratio increased from 15% to 25%, the reconstruction error decrease tended to be smooth. It can be seen that, in the five preset values, the compression rate and reconstruction error of the extracted key frames were relatively reasonable when the preset compression rate is 15%.

#### 3.3.2. Differences in Motion Accuracy on Key-Frames

The differences in motion accuracy on key-frames between novice and senior students are shown in Table 9 and Table 10.

From the results of the key-frames on clustering, the motion accuracy of the eight motions of novice and senior students were significantly different. This result is consistent with the result based on the original frames. However, on the key-frames of inter-frame pitch on five different thresholds, there was no significant difference in motion accuracy between the two groups in Motion 7. 

**Figure 7:**
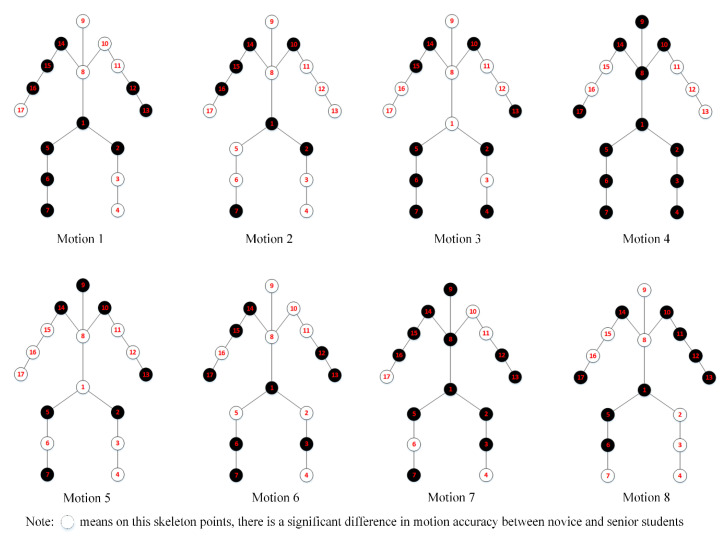
Differences in motion accuracy of points between novice and senior students on the key-frames of inter-frames pitch (Threshold = 1).

The differences in motion accuracy of points between the two groups on key-frames were also evaluated. Figure 7 shows the results on the key-frames of inter-frame pitch when the setting threshold = 1. 

**Figure 8:**
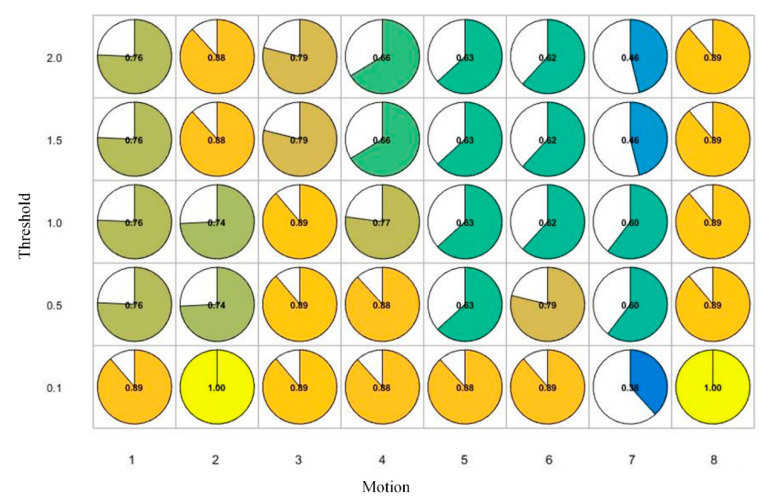
The Kendall coefficient of differences between skeleton points based on two difference methods (on the original frames and the key-frames on inter-frame pitch).

**Figure 9:**
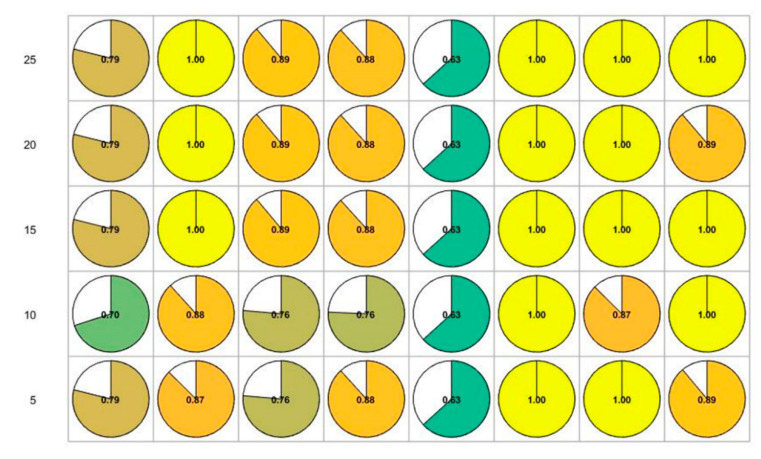
The Kendall coefficient of differences between skeleton points based on two difference methods (on the original frames and the key-frames on clustering).

From Figure 6 and Figure 7, we find that there was a difference between the results on the original frame and the key-frames on the inter-frame pitch. When there was a significant difference in motion accuracy between the two groups, we set the point to 1, otherwise, it was 0. Then, we evaluated the correlation between the results on the original frames and different key-frames on the Kendall correlation coefficient test (see Figure 8 and Figure 9). 

The results for key-frame extraction on inter-frame pitch show that when the threshold value was 0.1, the result of the differences in motion accuracy on the key-frames was highly correlated with the result based on the original frame (Kendall coefficient of points in each motion is higher than 0.7 except for Motion 7). However, when the threshold was 0.1, the compression rates of the key-frames were higher. As shown in Table 7, when the threshold was 0.1, the compression rate of each motion exceeded 50%. For key-frames extraction on clustering, there is a high correlation when the compression rate is 0.1. The Kendall coefficient of points in each motion is higher than 0.7 except for Motion 5, where the coefficient was 0.63. 

We also tested the mean processing time for using DTW to calculate the distances between motions on original frames and key-frames (Table 11).

From Table 11, the processing time on the key-frames is lower than original frames. Therefore, using key-frames can effectively decrease data processing time.

## 4. Discussion

When using mathematical methods, the macro differences between the motion data of novice students and the teacher were higher than the distances between the motion data of senior students and the teacher on eight motions of Baduanjin. Because the motion data of the experimental analysis are the rotation data of specific skeleton points measured by the IMU, if the teacher’s motions were taken as the standard, the results show that the motions of senior students were closer to the standard motions. Therefore, IMU can effectively distinguish the differences in motion accuracy in Baduanjin between novice and senior students. 

When using the original frames to evaluate the differences at 17 skeleton points in eight motions between novice and senior students, the results show the differences in motion accuracy between the two groups on skeleton points varied for the different motions. For Motion 1, the differences between the two groups were mainly concentrated on the head-spine segment and upper limbs, especially the right upper limb. The differences mean that the motion errors of novice students relative to senior students were mainly concentrated on these joints. The results are consistent with the common motion errors described in the official book: “When holding the palms up, the head is not raised enough, or the arms are not raised enough” [30]. However, for Motion 4, the common motion errors are described in the official book as: “Rotating head and arm are insufficient” [30]. The description shows that the main errors occur in the head-spine and bilateral upper limbs. However, significant differences of skeleton points were at bilateral upper limbs but not head-spine. This difference may be related to the small number of participates in this study. 

In this study, we also used two methods to extract key-frames. The raw data can be effectively compressed to decrease the data storage space using extracting key-frames [40,41]. The repetitiveness of action exercises in the teaching process will generate an extremely large amount of raw data. From the results, both key-frames extraction methods can effectively compress the raw data. We also found that the data processing speed could be accelerated on key-frames. However, the compression rates of key-frames on different motions when using key-frames on inter-frame pitch were different. We found that the differences in skeleton points on the key-frames on inter-frame pitch were not consistent with the results on the original frames. However, there was high consistency between the results on the key-frames on clustering and the results on the original frames, especially when the compression rate was 15%. Therefore, we can use key-frames to replace the original frames to evaluate motion accuracy of Baduanjin in order to decrease data storage space and processing time.

However, the small number of participants in our study limits the application of the results. As the participants were from a university in China, the results might only be suitable for university students in China because different populations have variations in anatomical characteristics, physiological characteristics, and athletic ability.

Based on our results, IMU can effectively distinguish the difference in the motion accuracy of Baduanjin between novice and senior students. Therefore, in the following work, we can develop a system using IMU to evaluate the motion quality of students and provide feedback to teachers and students. Thus, it would be able to assist teachers in correcting errors in the motions of students immediately.

## 5. Conclusions

These initial results show that, based on the original frames, the IMU and the corresponding mathematical methods can effectively distinguish the motion accuracy of all eight motions of Baduanjin between novice and senior students. Furthermore, the IMU can identify the differences between the novice and senior students on the specific skeleton points of the eight motions of Baduanjin. The results regarding key-frames on clustering were highly correlated with the results of the original frames, which means, to a certain extent, that key-frames can replace the original frame to decrease the data storage space and processing time.

## Figures and Tables

**Table 1:** The weightage of the 17 skeleton points.

Point	Weightage
Hip	16
Right up leg	8
Right leg	4
Right foot	2
Left up leg	8
Left leg	4
Left foot	2
Spine	8
Head	4
Right shoulder	4
Right arm	2
Right forearm	1
Right hand	0.5
Left shoulder	4
Left arm	2
Left forearm	1
Left hand	0.5

**Table 2:** Demographic characteristics of the students.

Group	Gender	Age (years) (mean ± SD)	Height (cm) (mean ± SD)	Weight (kg) (mean ± SD)
Novice Student	Male: 5	18.60 ± 0.55	169.40 ± 3.91	58.20 ± 4.60
	Female: 4	18.75 ± 0.96	161.25 ± 3.59	48.25 ± 2.06
Senior Student	Male: 4	20.25 ± 0.50	170.75 ± 4.11	60.50 ± 4.80
	Female: 7	20.00 ± 0.82	161.43 ± 3.84	48.57 ± 3.74

**Table 3:** Mean duration of Baduanjin.

Motion	Valid	Mean duration (s) (mean ± SD)
Motion 1	63	12.24 ± 2.49
Motion 2	63	21.52 ± 4.19
Motion 3	63	16.75 ± 4.14
Motion 4	63	14.79 ± 3.85
Motion 5	63	19.18 ± 5.25
Motion 6	63	16.95 ± 4.09
Motion 7	63	13.10 ± 3.93
Motion 8	63	1.51 ± 0.25

**Table 4:** Normality of data of groups using the Shapiro-Wilk test.

Motion	Group	Statistic	df	Sig.
1	Novice Student	0.788	27	0.000
	Senior Student	0.753	33	0.000
2	Novice Student	0.962	27	0.408
	Senior Student	0.973	33	0.575
3	Novice Student	0.956	27	0.299
	Senior Student	0.948	33	0.120
4	Novice Student	0.963	27	0.428
	Senior Student	0.979	33	0.758
5	Novice Student	0.963	27	0.428
	Senior Student	0.979	33	0.758
6	Novice Student	0.789	27	0.000
	Senior Student	0.852	33	0.000
7	Novice Student	0.881	27	0.005
	Senior Student	0.878	33	0.001
8	Novice Student	0.932	27	0.078
	Senior Student	0.890	33	0.003

**Table 5:** Differences in motion accuracy between novice and senior students on original frames (using the independent sample T-test).

Motion	Group	N ^1^	Mean ^2^	Std. Deviation	F	Sig.	t	Sig. ^3^
2	Novice Student	27	640.76	74.38	2.289	0.136	4.275	0.000
	Senior Student	33	565.72	61.64			4.195	0.000
3	Novice Student	27	543.46	78.92	4.879	0.031	5.085	0.000
	Senior Student	33	455.75	54.30			4.903	0.000
4	Novice Student	27	536.45	41.44	0.061	0.806	5.805	0.000
	Senior Student	33	468.66	47.70			5.888	0.000

^1^ Number of motions; ^2^ Mean of differences in motion between teacher and students; ^3^ 2-tailed.

**Table 6:** Differences in motion accuracy between Novice students and senior students on original frames (using the Mann-Whitney U test).

Motion	Group	N ^1^	Mean Rank	Sum of Ranks	M-W U ^2^	Wilcoxon W	Z	Asymp. Sig. ^3^
1	Novice Student	27	38.52	1040.00	229.00	790.00	−3.217	0.001
	Senior Student	33	23.94	790.00				
5	Novice Student	27	41.96	1133.00	136.00	697.00	−4.599	0.000
	Senior Student	33	21.12	697.00				
6	Novice Student	27	35.93	970.00	299.00	860.00	−2.177	0.029
	Senior Student	33	26.06	860.00				
7	Novice Student	27	37.41	1010.00	259.00	820.00	−2.771	0.000
	Senior Student	33	24.85	820.00				
8	Novice Student	27	42.19	1139.00	130.00	691.00	4.688	0.000
	Senior Student	33	20.94	691.00				

^1^ Number of motions; ^2^ Mann-Whitney U; ^3^ 2-tailed.

**Table 7:** Compression rate and reconstruction error of corresponding key-frames on inter-frame pitch.

Threshold	Index	Motion							
		1	2	3	4	5	6	7	8
0.1	Rate ^1^	60.45	80.57	55.84	62.00	86.84	76.58	58.83	67.30
	Error ^2^	0.059	0.028	0.062	0.046	0.017	0.042	0.022	0.068
0.5	Rate	15.28	27.36	14.02	16.87	36.75	25.12	15.29	20.16
	Error	0.447	0.364	0.455	0.378	0.330	0.448	0.474	0.612
1.0	Rate	7.74	14.79	7.09	8.77	20.72	13.39	8.03	9.97
	Error	1.031	0.904	1.110	0.971	0.799	1.021	1.164	1.969
1.5	Rate	5.14	10.04	4.70	5.93	14.44	9.05	5.57	6.36
	Error	1.811	1.590	1.967	1.719	1.346	1.692	2.099	3.971
2.0	Rate	3.81	7.58	3.49	4.48	11.12	6.80	4.35	4.50
	Error	2.712	2.362	3.021	2.586	1.966	2.474	3.203	6.936

^1^ Compression rate (%); ^2^ Reconstruction error.

**Table 8:** Reconstruction error of corresponding key-frames on clustering.

Rate (%) ^1^	Reconstruction Error							
	Motion 1	Motion 2	Motion 3	Motion 4	Motion 5	Motion 6	Motion 7	Motion 8
5	4.528	7.185	3.875	3.430	8.790	6.823	3.886	6.206
10	1.484	2.428	1.268	0.997	2.927	2.359	1.281	2.216
15	0.851	1.244	0.638	0.547	1.485	1.286	0.650	1.342
20	0.498	0.757	0.401	0.353	0.971	0.797	0.415	0.769
25	0.366	0.518	0.281	0.235	0.700	0.569	0.297	0.531

^1^ Compression rate (%) of key-frames.

**Table 9:** Differences in motion accuracy on the key-frames on inter-frame pitch between novice and senior students.

Threshold	*p* Value							
	Motion 1	Motion 2	Motion 3	Motion 4	Motion 5	Motion 6	Motion 7	Motion 8
0.1	0.000	0.000	0.000	0.000	0.000	0.006	0.075 ^1^	0.000
0.5	0.001	0.000	0.001	0.005	0.000	0.004	0.122 ^1^	0.000
1.0	0.001	0.000	0.001	0.017	0.004	0.004	0.122 ^1^	0.000
1.5	0.001	0.000	0.002	0.050 ^1^	0.008	0.004	0.141 ^1^	0.000
2.0	0.001	0.001	0.004	0.112 ^1^	0.018	0.004	0.176 ^1^	0.000

^1^*p* ≥ 0.05.

**Table 10:** Differences in motion accuracy on the key-frames on clustering between novice and senior students.

Rate (%) ^1^	*p* Value							
	Motion 1	Motion 2	Motion 3	Motion 4	Motion 5	Motion 6	Motion 7	Motion 8
5	0.000	0.000	0.000	0.000	0.000	0.001	0.003	0.000
10	0.000	0.000	0.000	0.000	0.000	0.024	0.003	0.000
15	0.000	0.000	0.000	0.000	0.000	0.020	0.004	0.000
20	0.000	0.000	0.000	0.000	0.000	0.031	0.004	0.000
25	0.000	0.000	0.000	0.000	0.000	0.024	0.004	0.000

^1^ Compression rate (%) of key-frames.

**Table 11:** The mean processing time on the original frames and the key-frames.

Motions	The Mean Processing Time (s)		
	Original Frames	Key-Frames ^1^	Key-Frames ^2^
1	1.891	0.021	0.042
2	5.960	0.195	0.180
3	3.674	0.026	0.078
4	4.439	0.027	0.069
5	5.515	0.218	0.117
6	4.055	0.053	0.069
7	2.209	0.028	0.043
8	0.145	0.013	0.017

^1^ Key-frames on inter-frames pitch (Threshold = 1); ^2^ Key-frames on clustering (compression rate = 15%).
